# On the Anthelmintic Action of Sulphate of Quinia

**Published:** 1855-08

**Authors:** Prosper Delvaux

**Affiliations:** Fellow of the Faculty of Medicine of the University of Brussels


*On the Anthelmintic Action of Sulphate of Quinia.* By Dr. PROSPER DELVAUX, Fellow of the Faculty of Medicine of the University of Brussels.

During the last three years I have proved the efficacy of sulphate of quina as an anthelmintic. Some days after the administration of this salt in the intermittent fevers of children, the parents came to announce to me the cure of the fever; but in a great number of cases, the little patients had had motions, followed by the expulsion of intestinal worms.

Thenceforth I asked myself, if sulphate of quinia had not anthelmintic properties? I then gave it to children affected with worms, and my trials were crowned with success. Every time that I made use of this salt as an anthelmintic it produced this effect, which I have verified in a great number of cases.

So early as 1764, Professor Van Doeveren of Groningen, reported two very interesting observations on the anthelmintic property of Peruvian bark.

The first case was that of a child, aged 12, affected with tænia. Purgatives, calomel, assafœtida, &c., had been tried, but in vain. At last, an ounce and a half of Peruvian bark was given in four days. After having taken the powder, the patient passed an entire tænia with the head.

In the second case, the patient was a young girl laboring under fever. She took an ounce of powdered Peruvian bark, made into an electuary with simple syrup. Three round worms were expelled.

In a great many cases, adds Van Doeveren, physicians have given this febrifuge with the single idea of subduing fever, and without having the least suspicion of worms, and nevertheless it has brought them away. Heister combined bark with mercury in his anthelmintic electuary, probably because he suspected the vermifuge power of the former.

I have collected upwards of forty cases of children affected with lumbricoid ascarides who have been radically cured of this affection with sulphate of quinia. The salt usually produces, at the end of twenty-four or thirty-six hours, several liquid motions containing these entozoa.

The sulphate of quinia is also most effectual in removing the ordinary ascarides (oxyures vermiculares.) As is well known, these parasites are lodged in the fæcal matters in the rectum, sometimes in the colon. They appear to imbibe a remnant of the chyle which serves to nourish them. They are expelled in a ball (peloton) with the fæces, or escape by themselves, causing intolerable itching, tenesmus, and other annoyances.

Injections of sulphate of quinia, repeated every evening for a certain time, are capable of completely destroying these entozoa.

I had twice an opportunity of administering sulphate of quinia for tænia, and in both cases the worm was expelled and was not reproduced. The first case occurred in October, 1855, and was that of a widow, aged 28, who had suffered for many years from a tænia of which she was constantly passing one or more segments. Every known anthelmintic had been administered without completely freeing her from her malady. After having taken about forty-six grains of sulphate of quinia, she voided several yards of a tænia, the characters of which corresponded with those of the bothriocephalus latus. The medicine was continued for some time, and she has ever since enjoyed good health, and has had no return of the worm attacks.

The subject of the second case, dating from the month of March, 1855, was a little boy, aged four, who, according to the report of the parents, had been from the time he was one year old in the constant habit of passing entire ells of a large flat worm, which I recognized to be the tænia lata. Sulphate of quinia was exhibited, a worm twenty-nine and a half feet (nine metres) in length was expelled, since which there has been no return of the affection.

Sulphate of quinia is, therefore, truly an anthelmintic. The physician often meets debilitated sickly young children, whose constitution bears the stamp of the most profound asthenia. He generally shrinks, when these children are at the same time attacked with worm affections, from the long list of anthelmintics, which, most frequently, only act on the digestive tube by producing violent effects, which are often felt injuriously throughout the entire system.

It is in such cases especially that the sulphate of quinia is advantageous, and I have never seen its employment followed by unfavorable consequences. Sulphate of quinia produces its vermifuge effects in virtue of its bitter properties; for bitters, as is well known, act more energetically as poisons on animals, in proportion as the latter are lower in the scale of creation.

Is it not on account of their bitter properties that Celsus and Cælius Aurelianus extol worm-wood and centaury as anthelmintics, and that Riviere (Praxis Medica, Book v. chap. 9,) praises the same and other plants as vermifuges, and as especially efficacious in removing lumbricoid ascarides? Kluyskens, in his treatise on "Materia Medica and Therapeutics," says that "bitters a:e very detrimental to worms." "It is a very curious fact," observes this writer, "that vegetable bitters should in general be so destructive to inferior animals; flies perish almost immediately on being wet with an infusion of quassia."

It is, therefore, not impossible that sulphate of quinia should be capable of killing intestinal worms. Moreover, by its tonic action, it restores the power of the digestive organs, debility of which strongly predisposes to the production of entozoa.

*Doses and Mode of Administration.*—The dose of the sulphate must vary according to the age of the patient; from two to ten years it will range between three and six grains; in older persons, so much as nine grains may be given in the twenty-four hours. When the medicine has produced the desired effect, the dose ought to be gradually diminished. During its administration the diet should be light. It is seldom necessary to have recourse to aperients; it may, however, occasionally be advisable to administer castor-oil or syrup of rhubarb. The following formulæ for its exhibition have been employed:—*Powders.*—Sulphate of quinia, half a scruple; sugar, as much as may be sufficient; divide into six powders. *Pills.*—Sulphate of quinia, half a scruple; honey, marshmallow powder, of each as much as sufficient; make into six pills. *Electuary.*—Sulphate of quinia, from three to six grains; purified honey, two ounces; mix; a teaspoonful to be taken frequently. *Syrup.*—Sulphate of quinia, from three to five grains; syrup of orangepeel, ten drachms; a teaspoonful to be taken at a time, and frequently repeated. *Enema.*—Sulphate of quinia, five or six grains; dilute sulphuric acid, as much as may be sufficient; distilled water, eight ounces.—*Presse Medicale Belge*, April 15, 1855, p. 125.

